# The impact of premature birth and low birth weight on motor, visual, and cognitive skills and mental health in adolescence: a systematic review and meta-analysis

**DOI:** 10.1007/s00787-025-02937-w

**Published:** 2026-01-14

**Authors:** Frederike Schröpfer, Emilia Greif, Sofia Eickhoff, Hannah Schewe, Jule Wiese, Nicole Derner, Luise Heller, Christina Andreou, Katharina Röse, Jonas Obleser, Wolfgang Göpel, Stefan Borgwardt, Léon Franzen

**Affiliations:** 1 Department of Psychiatry and Psychotherapy, University of Lübeck, Lübeck, Germany; 2 Center of Brain, Behavior and Metabolism (CBBM), University of Lübeck, Lübeck, Germany; 3 Institute of Health Sciences, Department of Occupational Therapy, University of Lübeck, Lübeck, Germany; 4 Department of Psychology, University of Lübeck, Lübeck, Germany; 5 Department of Neonatology, University of Lübeck, Lübeck, Germany

**Keywords:** Preterm birth, Low birth weight, Adolescence, Cognition, Mental health, Neurodevelopmental outcomes

## Abstract

Mental disorders represent a significant challenge for individuals and society. Many of them have a detectable onset during adolescence. Being born preterm or with low birth weight (PTB) has been emerging as a potential risk factor for developing mental health disorders in adolescence. Since PTB infants are considered to be at an increased risk of cognitive and sensory difficulties and are at risk for visual impairments, this systematic review aims to explore (1) whether evidence for a possible interplay between PTB, cognitive, visual, and motor abilities exists in the literature, and (2) whether and how these factors may relate to mental health outcomes of PTB individuals in adolescence. We conducted a registered systematic review following the PRISMA guidelines (PROSPERO #42024513150). The search strategy focused on the databases PubMed, Scopus, PsycINFO and the Cochrane Library and included publications sampling participants born in 1980 or later. Upon screening 499 studies, we analysed 17 studies including a total of 10,842 adolescents aged 11–20 (PTB = 8,813, control = 4,029) published between 2005 and 2022. PTB adolescents exhibited deficits in cognitive and motor domains compared to their full-term peers (≥ 37 gestational weeks; FT), including lower intelligence quotient (IQ), attention and executive function, and motor control. These effects can persist into adolescence and even adulthood. Importantly, several studies demonstrated that PTB adolescents receive diagnoses of psychiatric disorders more often and get diagnosed with more complex psychiatric disorders. Contrarily, evidence for subtle visual alterations and direct links between PTB, the reviewed domains and mental health outcomes remains scarce. These findings highlight that PTB adolescents can face challenges across multiple separate domains, including an elevated prevalence of psychiatric diagnoses. Clarifying the nature of these observational relationships shall provide insights that could improve early detection approaches and targeted intervention in the future.

## Introduction

Mental well-being among adolescents in the WHO European Region declines with increasing age: With most mental disorders having a detectable onset around age 15 [1], it is during late adolescence and early adulthood that most mental disorders become clinically detectable [2–4]. Their first signs, including subtle cognitive, psychological, and physical changes, may already appear during prodromal phases up to five years prior, as is the case with psychotic disorders [2]. Hence, prodromal phases may already fall into the early stages of adolescence. Mental ill-health contributes to at least 45% of the disease burden in adolescents and is often met with stigma and limited access to care. If a mental disorder begins in early adulthood, affected individuals may experience a reduction in life expectancy of 10–20 years [5].

Adolescence, defined by the WHO as ages 11 to 20 [3], marks a critical transition from childhood to adulthood and involves rapid biological, neural, and psychological changes [4, 6]. Current literature suggests it to be a critical time period for the early detection of mental ill-health, which increases the importance of the entire window from conception to early adulthood for understanding potential changes and risk factors.

Successful early detection of mental health problems heavily depends on the identification of the most informative risk factors. One risk factor may be being born preterm (< 37 weeks of gestation) or with very low birth weight (< 1500 g at birth), henceforth PTB. Globally, an estimated 15 million babies—more than 1 in 10—are born preterm each year [7, 8]. PTB is increasingly recognised as a key factor that may influence later neurodevelopmental outcomes and mental well-being [6]. PTB individuals face a 3- to 4-fold risk of developing one mental disorder later in life [9]. For example, schizophrenia is likely caused by an interplay of genetic and environmental factors that may include pre- and perinatal adversity, suboptimal postnatal environments in infancy and childhood, biological, psychological, and social stressors in adolescence [3, 10, 11].

PTB individuals are also at an increased risk of exhibiting cognitive, neuromotor or visual deficits later in life [6, 12, 13]. For instance, there is a significant risk that these individuals will suffer from reduced visual acuity. In severe cases, this may result in the development of cerebral visual impairment (CVI), or retinopathy of prematurity (ROP). Those substantial visual impairments due to the latter have been reported to contribute to psychomotor delays [14]. In general, accurate vision and perception are paramount for navigating daily life, as the visual modality provides the richest information of all our senses [15]. In sighted individuals, it provides context to all the other senses and enables the construction of multi- or supramodal representations and predictions [15]. If this context is removed, it can elicit symptoms of psychosis, such as hallucinations, even in healthy individuals [16]. The visual sense also supports the development of motor abilities for navigating and interacting with our world [17].

In addition, PTB children show poorer development of both fine and gross motor skills, which often persist into adulthood [18]. Poorer motor skills have been associated with higher levels of psychiatric symptoms and lower quality of life scores, underlining the importance of examining both visual and motor skills in relation to mental health [19].

Regarding cognition, some studies have operationalised cognitive outcomes as IQ scores. On average, PTB individuals tend to score lower on standardised IQ tests than their FT peers, with mean differences of approximately 12 points reported in certain studies [20]. In some cases, cognitive difficulties appear to persist into later childhood and adolescence, with up to 50% of affected children still exhibiting such patterns at age 11 [9], and potentially extending into adulthood [20]. The importance of IQ scores is underlined by scores from 2-year-olds being predictive of IQ outcomes in adulthood [21]. These differences are not surprising, since most of the brain development occurs in the third trimester of pregnancy [22]. Meaning that PTB individuals are born during or before this important and vulnerable period for brain development, which might give them a disadvantage from the starting line.

Although researchers and clinicians are aware of the outlined differences and factors related to PTB and specific birth circumstances, first studies report that the predictive accuracy of preterm events and factors favouring the development of a psychiatric disorder remains low [23, 24]. This low predictive accuracy might be attributed to relying on self-reports and examining already help-seeking individuals, which is a common limitation of the indicated prevention paradigm [2]. The focus on proximal predictors, such as functional decline and attenuated psychotic symptoms, may have led to an underrepresentation of distal risk factors like PTB [23].

Clear evidence for the role of PTB in psychiatric vulnerability comes from birth cohort studies [25]. A study examining adults born very premature (i.e., before 33 weeks of gestation) found higher rates of psychiatric symptomatology, including increased positive, negative, cognitive, and behavioural symptoms, compared to FT-born adults [24]. These individuals were significantly more likely to fall into a high-risk psychopathology cluster, indicating a general psychiatric vulnerability rather than a disorder-specific risk [24]. These findings align with the neurodevelopmental model of psychiatric disorders, postulating that altered brain connectivity in preterm individuals may contribute to their increased susceptibility to develop mental ill-health later in life [24].

Current approaches to early detection are predominantly informed by indicated prevention, with limited consideration given to the specific context of birth and psychometric assessments of low-threshold markers, such as visual and motor function. Indicated prevention may overlook subtle early signs in these low-threshold indicators, which can sometimes be observed well before help-seeking behaviour begins. The integration of existing prevention paradigms, namely indicated and selective prevention, might hold potential for enhancing early detection and overall health outcomes for individuals with PTB [2]. However, to date, a synthesis of potential concomitant developmental links between visual, motor, and cognitive skills after PTB and the risk of developing mental health problems in adolescence is lacking [24]. Therefore, the present review aims to address this gap by systematically examining (1) whether evidence for a possible interplay between preterm birth and cognitive, visual, and motor abilities exists, and (2) whether and how these factors may relate to mental health outcomes of PTB individuals in adolescence.

## Methods

The present systematic review was conducted according to the Preferred Reporting Items for Systematic Reviews and Meta-Analysis (PRISMA) guidelines [26]. The protocol was registered in the International Prospective Register of Systematic Reviews (PROSPERO) database on6/04/2024 (registration #42024513150).

### Literature search

A comprehensive literature search was conducted using multiple databases, including PubMed, Scopus, PsycINFO and the Cochrane Library, with a cut-off date of 6th November 2023. Handsearching of reference lists of relevant articles and systematic reviews supplemented the systematic search (done by FS and LF). Filters ensured the inclusion of papers written in English. Our search included randomised and non-randomised trials, and observational studies (cohort and case-control) meeting the inclusion criteria. We excluded animal studies, dissertations, books and book chapters, and opinion papers as well as articles without a representative sample size of preterm and/or low birth weight participants (*N* < 20). Duplicate records were removed. Preterm birth was defined as birth before 37 completed weeks of gestation, in line with the World Health Organisation classification [27].

### Study selection

The study selection process was managed using Covidence (www.covidence.org). At all stages reviewers (FS and LF) assessed articles for relevance to the research question and eligibility criteria. A two-step screening process was conducted, beginning with a title and abstract screening, followed by a full-text review to assess eligibility based on the predefined inclusion and exclusion criteria. The reviewers examined the texts to make final decisions based on predefined eligibility criteria. Disagreements between reviewers during this process were resolved by discussion amongst these two reviewers.

The search strategy was developed to capture studies linking PTB to mental health as well as motor, visual, and cognitive abilities in adolescents. We operationalised mental health in accordance with the WHO as an inclusive state of well-being [28] that goes beyond mere diagnostic categories in established classification systems. Motor abilities encompassed gross and fine motor domains and visual abilities focused predominantly on subtler functional aspects that excluded severe conditions such as CVI, ROP or blindness. For instance, studies including participants with CVI were excluded because CVI represents a heterogeneous group of neuro-visual disorders that complicate the differentiation of specific visual abilities and may mask subtler visual deficits. Inclusion and exclusion criteria were defined prior to data selection and extraction (Table 1). Articles were eligible if all participants were born on the 1 st of January 1980 or later, reflecting the impact of major advances in neonatal care, including surfactant therapy [29, 30], improved ventilators and incubators [31], the adaption of family-centred care models [32], and markedly increased survival rates for extremely preterm infants [31]. Further eligibility criteria were defined as being born preterm (< 37 weeks), and/or with low birth weight (< 2400 g), being at least 10 years old at the time of testing, and exhibiting an IQ score > 70. Studies involving participants with conditions like down syndrome or cerebral palsy or with prenatal exposure to frequent illicit drug or alcohol use in undifferentiated samples were excluded, following a similar logic to that used for CVI. To ensure methodological rigor, we focused on original observational studies and excluded reviews, editorials, and small-sample studies (< 20). To consider maximum information, we also included studies reporting results of a subsample fulfilling these criteria (e.g., [33, 34];Table 2).

**Table 1:** Inclusion and exclusion criteria for study selection. The criteria were applied to identify eligible studies assessing motor, visual, and/or cognitive abilities in PTB adolescents, in relation to mental health outcomes

Criterion	Inclusion Criteria	Exclusion Criteria
Sample birth year	Participants born in January 1980 or later	—
Gestational age	Preterm (< 37 weeks gestation)	—
Birth weight	Low birth weight (< 2400 g)	—
Assessed abilities or outcomes	Motor, visual, and/or cognitive abilities, and/or mental health after PTB	—
Age at testing	≥ 10 years old at the time of testing	—
Study types	Observational studies (cohort, case-control), randomised trials, perspective articles	Reviews, book chapters, editorials, opinion papers, small samples (< 20)
IQ threshold	Absence of intellectual disability (IQ > 70)	—
Excluded conditions	—	Down syndrome, cerebral palsy, PVL, grade IV IVH
Excluded maternal factors	—	Maternal history of frequent illicit drug or alcohol use during pregnancy

**Table 2:** Search string on PubMED

((“mental health“[MeSH Terms] OR “mental health“[All Fields] OR “mental disorder“[All Fields] OR “mental illness“[All Fields] OR “mental disorders“[MeSH Terms] OR “psychotic disorders“[MeSH Terms] OR “psychosis“[All Fields])
AND
(“preterm birth“[All Fields] OR “premature birth“[MeSH Terms] OR “low birth weight“[All Fields] OR “low birth weight infant“[All Fields])
AND
(“motor skills“[MeSH Terms] OR (“motor“[All Fields] AND “skill*“[All Fields]) OR “visual perception“[MeSH Terms] OR (“visual“[All Fields] AND “skill*“[All Fields]) OR “cogniti*“[All Fields])
AND
“adolescen*“[All Fields])

**Fig. 1:**
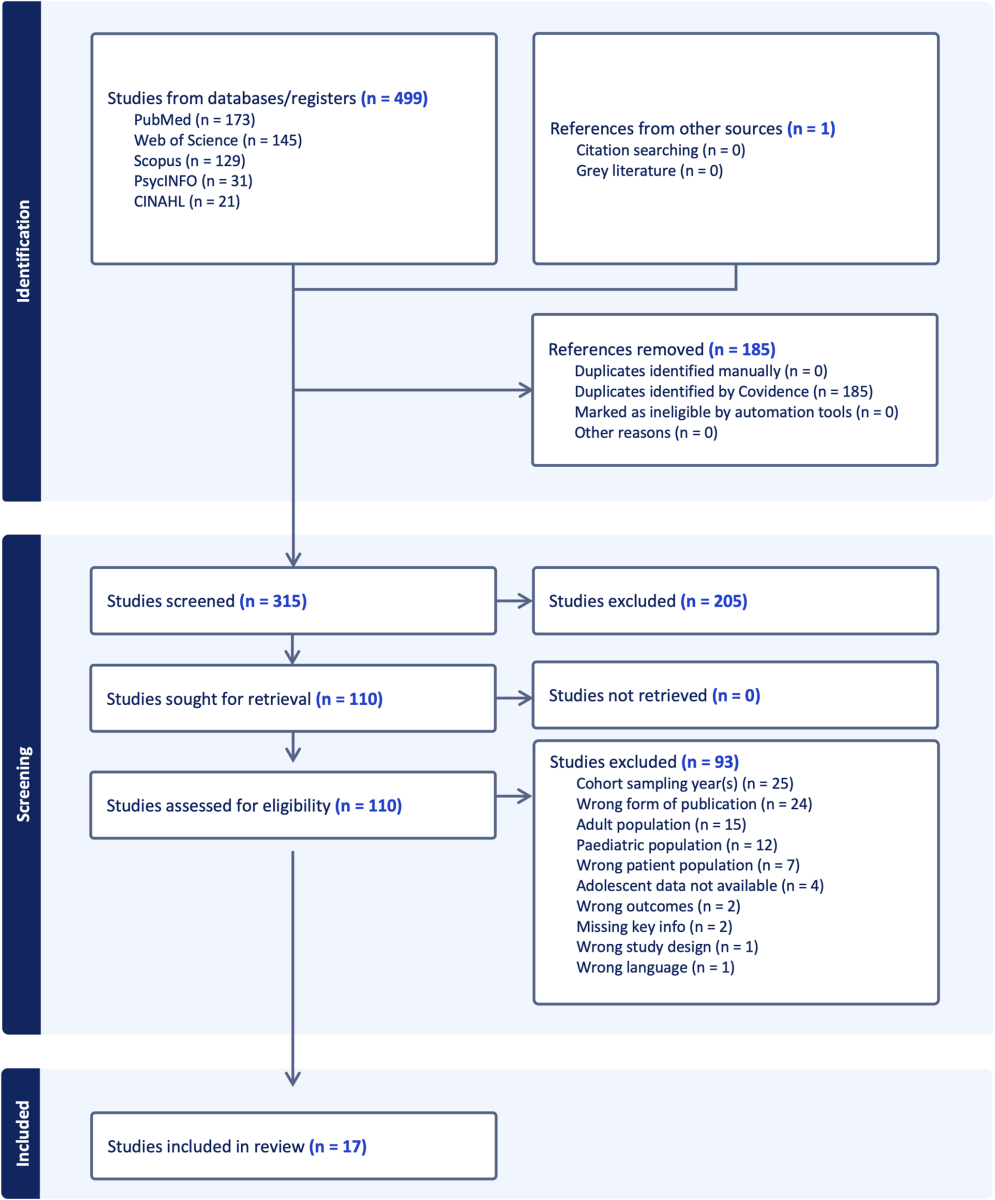
PRISMA-Flowchart of the study selection process. Flowchart of study selection based on PRISMA guidelines. Studies were excluded for the following reasons: cohort sampling years not matching criteria (*n* = 25), wrong form of publication (*n* = 24), adult population (*n* = 15), paediatric population (*n* = 12), adolescent data not available (*n* = 4), wrong patient population (*n* = 7), wrong outcomes (*n* = 2), missing key information (*n* = 2), wrong study design (*n* = 1) and wrong language (*n* = 1)

The initial literature search identified a total of 499 studies across all databases (Fig. 1). Of these, we excluded 185 duplicates. The remaining 315 studies were screened by two independent reviewers (F.S. and L.F.) using title and abstract screening, which resulted in the exclusion of 205 studies. Subsequently, a total of 110 studies were subjected to full-text screening. We excluded 93 studies at this stage, as they did not meet all pre-registered inclusion criteria (Table 1).

This process resulted in a final sample of 17 articles analysed in this systematic review (Fig. 1; Table 3). Most included studies report results from individuals who had to be born before 30 weeks of gestation, with one study reporting on data obtained from younger preterms (23–25 weeks of gestation; [33]), and two studies reporting on data from slightly older preterms (32–37 weeks of gestation; [34, 35]). The birth weight of sampled individuals ranged between < 1000 and < 1500 g across all studies. Since we could not identify substantial differences in results relevant for our findings, we synthesise all studies using the acronym PTB.

**Table 3:** Table of findings

Paper	Type of Publication	Cohort (Characteristics)	*N* PTB (*N* Controls)	Estimates				Strobe Score
				Cognition	Motor Function	Visual Function	Mental Health	
Brylka et al. (2021) [34]	Individual participant data meta-analysis of two studies	Millennium Cohort Study (MCS) (≤ 32 weeks of gestation)	3182 (3137)				Hyperactivity/inattention *p* =.01	77.27%.
		Basel Study of Preterm Children (BSPC) (≤ 32 weeks of gestation)	99 (59)					
Burnett et al. (2015) [48]	Longitudinal cohort study	Victorian Infant Collaborative Study (VICS) (< 28 weeks of gestation)	394 (166)	CogState detection *p* =.09			Attentional control (HSCT) time *p* =.002	81.82%
							Cognitive flexibility *p* =.001	
				CogState identification *p* =.19			Goal setting (RCF) copy accuracy *p* =.001	
							Goal setting (RCF) organization score *p* =.001	
Frazier et al. (2022) [11]	Longitudinal cohort study	Extremely Low Gestational Age Newborns (ELGAN) Study (< 28 weeks of gestation)	670 (none)				♀:♂ *p* <.05 major depressive disorder, ADHD hyperactivity	86.3%
							♀:♂ *p* <.01 generalised anxiety disorder, separation anxiety disorder, social anxiety disorder	
							♀:♂ *p* <.001 agoraphobia	
Ganella et al. (2015) [51]	Longitudinal cohort study	Ganella Population **(**GANE-P) (< 28 weeks of gestation)	341 (147)	IQ Score (age 18): 96.8, 95% CI [6.0, 16.2], *p* <.001			Any mood disorder *n* = 32 (16%), *p* =.20	81.82%
							Any psychiatric diagnosis past or current *n* = 58 (30%), *p* =.10	
							Any ADHD diagnosis *n* = 29 (15%), *p* =.01	
Haebich et al. (2018) [50]	Longitudinal cohort study	Victorian Infant Brain Study (VIBeS) (< 30 weeks of gestation; < 1250 g birth weight)	238 (77)	Tower Test total achievement raw score *p* <.001	Copy raw score ‑2.39, 95% CI [‑3.61, ‑1.17], *p* <.001			90.91%
				IQ Score (age 13): 100.2, SD = 18.3				
Holsti et al. (2017) [33]	Longitudinal cohort study	Holsti Population (HOLS-P) (23–25 weeks of gestation)	237 (103)	Major cognitive impairment *p* <.001	Poor motor skills *p* <.001	Severe or moderate visual impairment *p* =.09	ADHD/ADD *p* =.002	95.45%
							Autism spectrum disorders *p* =.06	
Indredavik et al. (2005) [52]	Cross-sectional study (group comparison)	Indredavik -Population **(**INDR-P) (≤ 1500 g birth weight)	199 (83)				Relative risk for psychiatric disorders compared to controls VLBW mother reported OR = 6.1, 95% CI [2.3, 16.7] VLBW father rep. OR = 3.9, 95% CI [1.3, 11.3] SGA mother rep. OR = 3.2, 95% CI [1.1, 9.6] SGA father rep. OR = 1.0, 95% CI [0.3, 3.8]	87.1%
James et al. (2019) [35]	Longitudinal cohort study	Jame-Population (JAME-P) (< 37 weeks of gestation)	104 (104)	IQ Score (age 15): 104.72, SD = 12.36 (within sibling comparison *ß*=−0.20, *p* =.04, 95% CI [−0.40, −0.01], compared to unrelated control *ß*=−0.35, 95% CI [‑0.64, −0.06], *p* =.02)			ADHD symptoms *p* =.04 (within sibling comparison *ß*=*0.32,* 95% CI [−0.05, 0.58], *p* =.01; compared to unrelated control ß=0.64, 95% CI [0.17, 2.05], *p* =.04)	82.82%
Johnson et al. (2010) [9]	Longitudinal cohort study	EPICure (≤ 26 weeks of gestation)	460 (153)				Any DSM-IV clinical diagnosis *p* =.0001	86.36%
							Any ADHD diagnosis *p* =.005	
							Any autism spectrum disorder diagnosis *p* =.0001	
Joseph et al. (2016) [42]	Longitudinal cohort study	Extremely Low Gestational Age Newborns (ELGAN) Study (23–27 weeks of gestation)	889 (none)	DAS-II verbal distribution (*p* <.001): ≤ −2 SD 17% > –2 and ≤ -1 SD 19% +/- 1 SD 57% >1 SD 7%	Visuomotor Precision distribution (*p* <.001): ≤ −2 SD 21% > – 2 and ≤ −1 SD 35% +/- 1 SD 38% > 1 SD = 7			72.73%
				DAS-II nonverbal reasoning distribution (*p* <.001): ≤ −2 SD 15% > – 2 and ≤ −1 SD 24% +/- 1 SD 56% > 1 SD 5%				
Kelly et al. (2022) [44]	Longitudinal cohort study	Victorian Infant Brain Study (VIBeS) (< 30 weeks of gestation)	187 (55)	IQ score (age 13): 97.8, SD = 17.0; 95% CI [5.0, 15.0], *p* =.0003	Motor score − 1.5, 95% CI [‑2.5, ‑0.4], *p* =.006			100%
		Paediatric Imaging, Neurocognition, and Genetics (PING) (< 30 weeks of gestation)						
Linsell et al. (2018) [46]	Prospective cohort study	EPICure (< 26 weeks of gestation)	219 (153)	IQ score (age 11): 83.4, 95% CI [80.8, 85.9]				100%
			127 (64)	IQ score (age 19): 85.7, 95% CI [82.7, 88.8]				
Linsell et al. (2019) [47]	Longitudinal cohort study	EPICure (< 26 weeks of gestation)	209 (148)				Total difficulties score (age 11): 11.1, 95% CI [10.1, 12.1]	90.91%
			134 (86)				Total difficulties score (age 16): 11.9, 95% CI [10.6, 13.2]	
			117 (55)				Total difficulties score (age 19): 12.2, 95% CI [10.9, 13.5]	
Luu et al. (2011) [53]	Cross-sectional study (group comparison)	Multicentre Randomised Indomethacin Intraventricular Haemorrhage Prevention Trial (IVHPT) (< 1250 g birth weight)	337 (102)	Executive Function adj mean difference −1.3 to −2.0 on D-KEFS subscales; memory adj mean difference − 0.8 to −7.1			Clinically significant BRIEF scores OR = 4.2, 95% CI [1.6, 10.9]	58.6%
Marlow et al. (2021) [45]	Longitudinal cohort study	Extremely Low Gestational Age Newborns (ELGAN) Study (< 26 weeks of gestation)	Sample 1996: 112 (153)	IQ score (age 11): 82.7, SD = 18.4			Combined mod/severe 51%	100%
			Sample 2006: 176 (143)	IQ score (age 11): 81.4, SD = 19.2			Combined mod/severe 47%	
				Neurodevelopmental impairment 2006 vs. 1996 *p* =.32 Reading impairment 2006 vs. 1996 unadjusted = 0.56, 95% CI [0.32, 0.97], *p* =.04			2006 vs. 1996 *p* =.88	
Tilley et al. (2017) [43]	Longitudinal cohort study	Extremely Low Gestational Age Newborns (ELGAN) Study (< 28 weeks of gestation)	43 (none)	Six genes predict neurocognitive impairment				90.91%
Wilson-Ching et al. (2013) [49]	Longitudinal cohort study	Victorian Infant Collaborative Study (VICS) (< 28 weeks of gestation; < 1000 g birth weight)	198 (153)	Symbols correct *p* <.05, d = 0.24				95.45%
				Selective attention strings correct *p* <.001, d = 0.56				
				Shifting attention strings correct *p* <.001, d = 0.56				
				Divided attention score *p* <.05, d = 0.27				

### Data extraction and quality assessment

The data extraction process involved the first author extracting the following variables for each included publication, if available: (1) name of the study; authors and publication year; (2) study design; (3) population; (4) analysed assessments or diagnostics; (5) findings. To quantify a study’s quality, we conducted the Strengthening the Reporting of Observational Studies in Epidemiology analyses (STROBE; [36]). Additionally, for each publication, we extracted all available data on cognitive, motor and/or visual abilities as well as on mental health.

### Data synthesis

The data synthesis included all relevant data from the systematically identified and included studies. Given the wide variability in topics and data within the identified studies and domains, all reported findings are presented and compared in the context of the research question. We also conducted meta-analyses of the IQ scores available from the studies included in this systematic review. Additionally, it is important to acknowledge that within the cognitive domain, we report findings from pen and paper tests alongside isolated results from self-report questionnaires. The latter may include a subjective component.

### Meta analyses

The R packages meta, metafor, and ggplot2 were used for statistical analyses and visualisation. The figures illustrating the results of the meta-analyses (i.e., age-stratified comparisons of mean IQ scores and raincloud plot [37]) were created using RStudio (version 4.3.2) and MATLAB (version 2024b, The MathWorks Inc., Massachusetts, USA), respectively. Additional graphical adjustments were made for readability using Adobe Illustrator.

James et al. [35] provided no IQ value for the control group. To fill this gap in the meta-analyses, the missing values (i.e., means, standard deviations, sample sizes) were estimated by averaging across the available data for control groups in the rest of the reviewed studies. These estimates of a “general” control group were then used as a proxy for the missing control group in the study [35]. This imputation procedure is consistent with recommendations for handling missing data in meta-analyses when the number of missing values is small and the comparison groups can be assumed to be sufficiently homogeneous [38, 39]. This imputation was performed for the control group exclusively.

Together, the amended control data and PTB data formed the basis for two random effect models estimating (1) the standardised mean difference in IQ scores and (2) Hedges’ *g* as a measure of effect size. These random-effects meta-analyses were conducted using the restricted maximum likelihood (REML) estimator, based on k = 8 independent effect sizes. The meta-analyses were conducted on an aarch64-apple-darwin20 in RStudio (version 4.3.3; 2024-02-29, “Angel Food Cake”; [40]) using the metafor package (version 4.8.0; [41]). The effect size model’s fit was slightly better (AIC_IQ_ = 52.07, BIC_IQ_ = 51.96; AIC_*g*_ = 40.58, BIC_*g*_ = 40.48). We report results from both models for transparency, as they may serve as a basis for future comparisons with alternative model specifications (e.g., fixed-effect models) and other studies reporting IQ scores.

To examine the robustness of our findings and assess potential heterogeneity, we conducted a leave-one-out sensitivity analysis by iteratively excluding each study from the dataset used in the meta-analyses. The results indicated that no single case disproportionately influenced the overall conclusion on IQ differences.

## Results

**Fig. 2:**
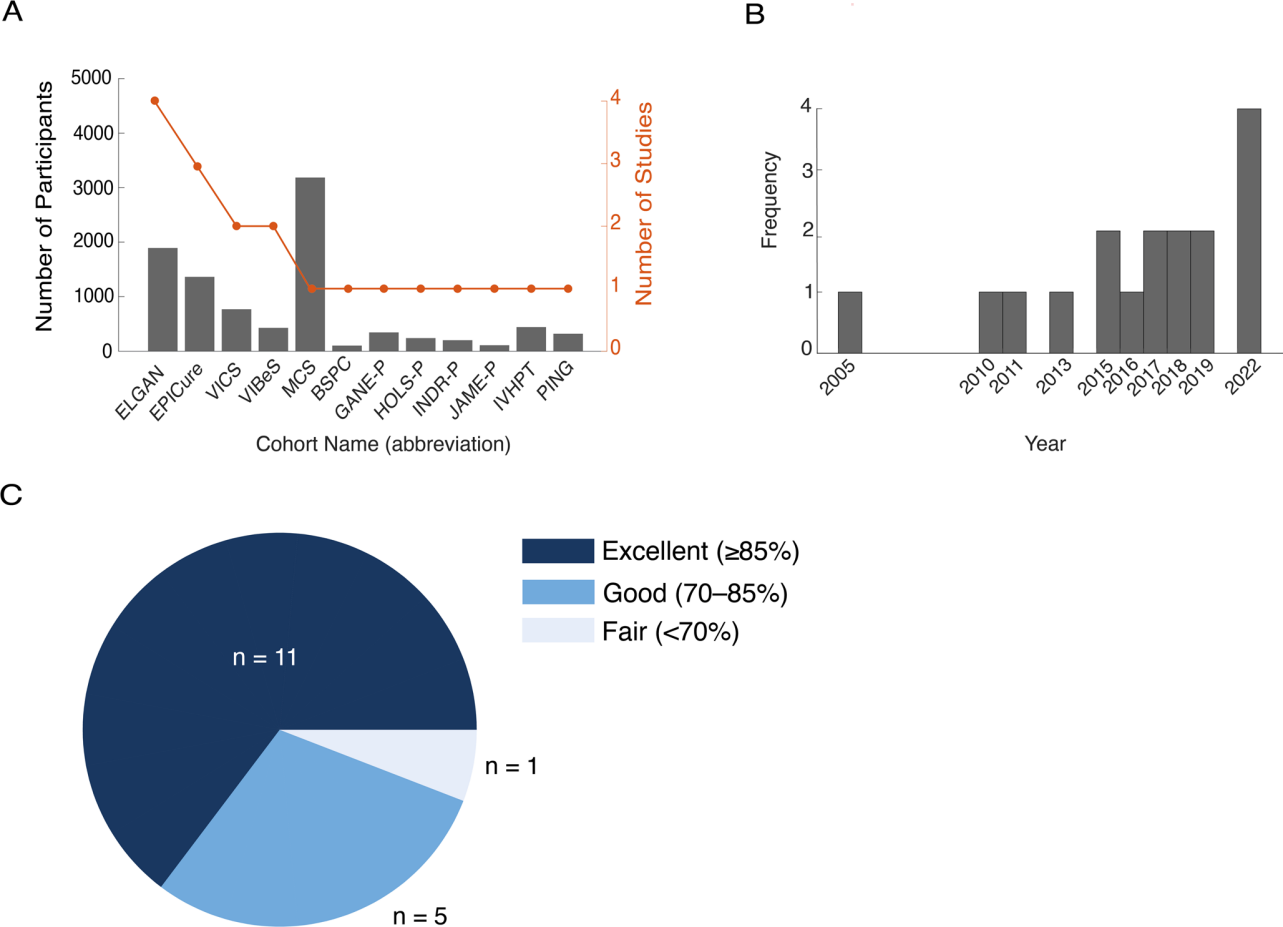
Number and cohort of included participants, temporal distribution, included publications.** (A)** Dual-axis plot displaying the number of participants and studies for each cohort, respectively (grey bars, left y-axis; red dotted line, right y-axis). The plot highlights differences between the cohorts’ sample size and the number of publications analysing their data. Bars represent the accumulated sample size per cohort. The red dotted line indicates the number of studies analysing each cohort’s data. **(B)** Histogram depicting the distribution of included studies by year of publication since 2005. These data indicate an increasing trend in the number of studies published over time with a very recent uptick in 2022. **(C)** Pie chart illustrating the distribution of STROBE quality scores (*n* = 11) rated as “Excellent” (≥ 85%), (*n* = 5) rated as “Good” (70–85%), (*n* = 1) classified as “Fair” (< 70%)

The present systematic review identified 17 eligible studies published between 2005 and 2022 (Fig. 2A). These studies included a total of 10,842 adolescents (PTB = 8,813, reported control = 4,029), three studies included no control participants [11, 42, 43]. Visual inspection shows that publication frequency has been increasing in recent years (Fig. 2B). According to STROBE, almost all studies were of excellent or good quality, with only one study receiving fair scores (Fig. 2C). These analysed data originate from several separate cohort studies, complemented by a few single original datasets (for an overview, see Table 1; Fig. 2A). Some studies analysed data from separate cohorts within the same publication [34, 44].

The included studies present a heterogeneous picture in terms of country of origin, participant age, and research team. The extremely low gestational age newborns cohort (ELGAN) appears in four included publications [11, 42, 43, 45]. The EPICure cohort is included in three publications [9, 46, 47]. The Victorian Infant Collaborative Study (VICS; [48, 49]) and the Victorian Infant Brain Study (VIBeS; [44, 50]) cohorts are each reported in two publications. Eight of the reviewed publications investigate a single cohort, or a cohort that is included in only one publication [33–35, 44, 51–53].

### Cognition

Eleven articles reported on cognitive abilities of PTB adolescents [33, 35, 42–46, 48, 50, 51, 53, 54]. Several studies examined IQ as a more detailed proxy of cognitive abilities, with IQ data included only once per cohort. The study by Marlow et al. [45] reports on data from two EPICure cohorts collected at two distinct time points throughout adolescence while the study by Linsell et al. [46] reports on the same cohort at two distinct time points.

**Fig. 3:**
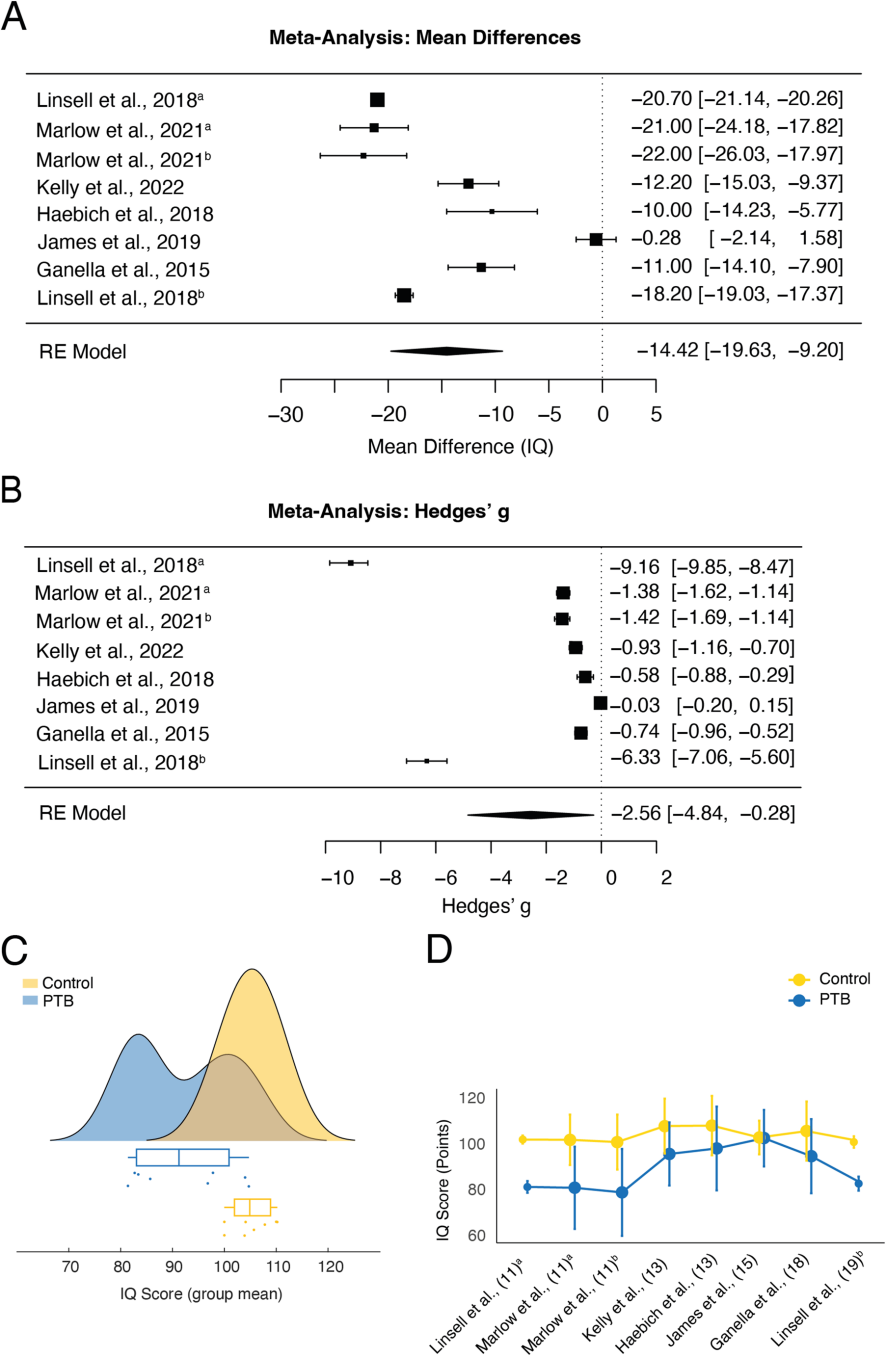
Meta-analyses of mean differences (MD) in cognitive IQ test scores and Hedges’ ***g*** across the reviewed studies.** (A)** Forest plot illustrating the MD in IQ test scores with their corresponding 95% confidence intervals for each examined study. Sorted by ascending age. Pooled effect sizes based on a random-effects model (*k* = 8; REML estimator). The overall reduction was statistically significant (*g* = −14.41, *SE* = 2.66, 95% CI [−19.63, −9.19], *p* <.0001). Between-study heterogeneity was substantial (τ² = 66.89; I² = 99.12%; Q(7) = 518.45, *p* <.0001), indicating considerable variation in effect sizes across studies. Model fit indices: AIC = 52.07, BIC = 51.96. Two studies report on two separate samples indicated by lower case superscript letters. Linsell et al., 2018ª assessed children at age 11, whereas Linsell et al., 2018ᵇ reported outcomes from the same cohort at age 19. Meanwhile, Marlow et al., 2021ª and Marlow et al., 2021ᵇ assessed two different samples of children both at age 11. **(B)** Forest plot illustrating Hedges’ *g* across reviewed studies with their corresponding 95% confidence intervals for each examined study. This list is sorted by ascending age. Pooled effect sizes based on a random-effects model (*k* = 8; REML estimator). Model fit indices: AIC = 40.58, BIC = 40.48. **(C)** Raincloud plot [37] of mean IQ scores depicted in panel A, and boxplots comparing scores between PTB (blue) and FT control samples (yellow) independent of age. **(D)** Comparison of mean IQ scores between PTB (blue) and FT control samples (yellow) sorted by age at the time of testing. Where no FT control data was available [13], we used the mean of the available control groups as a comparator

Our meta-analysis of IQ raw scores revealed a mean IQ score of 92.5 points of PTB adolescents across studies, with a mean difference of −14.44 compared to FT control groups (*k* = 8, *SE* = 2.77, 95% CI [−19.63, −9.20], *p* <.0001; Fig. 3A). An additional random-effects model of effect sizes corroborated this significant pooled reduction in IQ scores (*k* = 8, *g* = −2.56, *SE* = 1.16, 95% CI [−4.84, −0.28], *p* =.028; Fig. 3B). Between-study heterogeneity was substantial (τ² = 10.80, *SE* = 5.79, τ = 3.28). Importantly, the I² value of 99.83% indicates that nearly all observed variance is due to true heterogeneity rather than sampling error. Cochran’s Q-test for heterogeneity was statistically significant (Q(7) = 889.47, *p* <.0001), providing more evidence for differences in reported IQ outcomes among preterm populations reflecting meaningful between-study differences rather than random variation. Interestingly, the effect size of two PTB samples reported within the same study was substantially larger than the remaining ones ([46]; Fig. 3B). Although this study increases the observed overall effect, a clear reduction in the IQ of PTB adolescents would be evident still, as only two studies report a non-significant reduction (Fig. 3A, B).

Both meta-analyses show high heterogeneity between studies. Leave-one-out analyses revealed that the studies by Linsell et al. [46] and James et al. [35] were the most influential. James et al. [35] increased the mean differences, while Linsell et al. [46] increased the differences in Hedges’ *g* more than the other studies. The strongest effect was observed for the younger sample reported by Linsell et al. (Est_with_ = −2.56, Est_without_ = −1.61; [46]). However, most importantly, the overall result of substantial reductions in IQ scores did not change after excluding these studies one-by-one and heterogeneity remained high across analyses (I²_min_ = 97.66). Changes in general IQ average over time due to improvements in education and medicine are a conceivable explanation. However, we found no significant relationship between IQ differences and year of testing (*tau* = − 0.15, *p* =.75).

Further analyses of the temporal evolution of IQ differences throughout adolescence illustrated that IQ score differences follow a U-shape, with PTB individuals exhibiting larger reductions at age 11 (Fig. 3D, Δ = −18.87 points, *M*_PTB_ = 83.4, 95% CI [83.0, 83.8] [13, 46]), and 19 (Δ = −18.2 points, *M*_PTB_ = 85.7, 95% CI [82.7, 88.8]; [46]). IQ differences between PTB and their FT peers decrease towards mid-adolescence at age 13 (Δ = −10.0 points, *M*_PTB_ = 100.2, 95% CI [97.50, 102.90]; [44, 50] and 15 (Δ = −4.33 points, *M*_PTB_ = 104.72, 95% CI [103.04, 106.40.]; [55]).

Several studies also examined specific cognitive abilities, such as executive functions and attention. PTB adolescents exhibited intact planning skills at age 13 with a score of 9.9 out of 10 on the Delis-Kaplan Executive Function System (D-KEFS; [56]). On the contrary, adjusted mean differences on executive function tasks across all D-KEFS subscales indicated that deficits also exist in the PTB group, albeit at varying levels. On the one hand, the smallest deficit was observed in the Category Fluency subtest (*M* = 9.7, *SD* = 3.7), with 6.3% of children scoring two standard deviations below the norm (*aMD* = −1.3, 95% CI [−2.1, −0.5]). On the other hand, the most significant impairment was observed on the Colour-Word Inhibition subtest (*M* = 7.9, *SD* = 3.8), where 17.6% of children scored two standard deviations below the norm (*aMD* = −2.0, 95% CI [−2.8, −1.2]; [53]). As well, PTB adolescents exhibited significant planning and organisation difficulties on the self-rating-based Behaviour Rating Inventory of Executive Function (BRIEF; *β* = 2.5, 95% CI [1.2, 5.1]; [48, 57]). More cognitive performance measures showed significant impairments in selective, shifting, and divided attention (*p* <.001, *p* <.001, *p* <.05, respectively; [49]). Neurocognitive impairment could even be predicted by six genes, as observed in a study involving preterm-born individuals [43].

Taken together, these studies highlight cognitive challenges including general IQ, executive function, and attention in PTB adolescents [50, 58]. These are particularly prominent in the beginning and end of adolescence, and may persist in adulthood.

### Motor function

Motor functioning relies on integrating neurosensory input to interpret environmental cues and generate appropriate responses. Four included studies examined motor function with a sample size of more than 20 participants [33, 34, 42, 50]. Two studies found a considerable prevalence of general motor impairment in adolescence [33, 42]. Specifically, during earlier adolescence (i.e., age 10–15), one study showed a generally higher prevalence of poor motor skills in PTB adolescents without neurosensory difficulties using the M-ABC [33]. Also in early adolescence, the majority of PTB individuals aged 10 showed performance considerably below average on the visuo-motor precision task (21% of participants scored ≤ (−2) SD; 35% scored between > (−2) and ≤ (−1) SD; 38% scored between > (−1) and ≤ 1 SD; 7% scored > 1 SD; [42]), evidencing deficits in fine motor control. As well at age 13, visuo-constructive abilities, which require fine motor sketching skills amongst visual integration, indexed by the Rey Complex Figure Test (RCFT; [59]), showed a significant reduction in the raw copy score (*M*_PTB_ = 24.94, *SD* = 4.93, *M*_FT_ = 27.28, *SD* = 3.80; Δ = −2.39, 95% CI [−3.61, −1.17], *p* <.001; [50]).

As a proxy of general motor issues, PTB adolescents demonstrated consistently lower levels of physical activity, reporting an average of 10 min less activity per day than their FT counterparts [34]. More specifically, a group of PTB adolescents without neurosensory impairment (NSI) was significantly more likely to receive physical/occupational therapy, which indicates difficulties (*aOR* = 11.1, 95% CI = [1.4, 87.7], *p* =.02; [33]).

It is noteworthy that not all studies report motor deficits. Brylka and colleagues [34] did not find a higher likelihood of NSI, which includes severe motor impairments, in either the Basel Study of Preterm Children or the Millennium Cohort Study samples (*p* =.563, *p* =.197, respectively; [34]). Importantly, these PTB cohorts comprise fewer attentional and peer problems than expected, while particularly the Millenium Cohort Study comprises considerably more FT births simply to increase statistical power [34]. Together, this specific compilation of experimental groups may have reduced the likelihood of detecting true effects of motor impairments and biased results due to potentially unequal variances of the groups.

In sum, the analysed studies provide evidence for an increased prevalence of motor deficits and less physical activity in PTB adolescents. Though, the evidence for an increased risk of neurosensory impairments in the reviewed studies is not unequivocal.

### Visual function

Three of the included studies investigated the effects of PTB on functional visual abilities including visual perception, visuo-motor control and visuo-spatial planning in adolescence [33, 42, 50]. Subtler deficits in these visual abilities are not as salient and compromising as CVI or ROP and have received limited attention in the literature. The reviewed studies show that PTB adolescents have an increased risk of scoring between one and two standard deviations below the FT population mean on most visual tests [42]. Developing proficient visual abilities, such as visual perception and mental flexibility, are particularly challenging for PTB adolescents [42]. One study observed a significant trend towards the need of glasses in PTB adolescents (*aOR* = 3.4, 95% CI = [1.7, 6.8], *p* <.001), with a few exhibiting visual acuity below 20/200 without correction in their better eye [33]. Lastly, PTB adolescents were less likely to improve their visual conceptual organisation, even after adjusting for baseline group differences. This skill, originating from the RCFT [59], requires substantial visuo-spatial perception and planning abilities during copying of a complex figure [42, 60]. A gender difference was reported for visuo-spatial organisation skills, where especially PTB males showed inferior performance [50].

The evidence, albeit preliminary due to the small number of studies, suggests that PTB adolescents may also be at increased risk of suffering from subtler visual impairments in visual perception and visuo-spatial organisation compared to their age-matched FT peers.

### Mental health

Eleven studies have investigated neurodevelopmental disorders, cognitive impairment and mental health in PTB adolescents [9, 11, 33–35, 46–48, 51–53]. These studies cover mild to severe mental health issues. In accordance with the broad definitions provided by the World Health Organization [28] and Keyes [61], we use the term “mental health” to denote assessments ranging from well-being to psychiatric symptoms without exclusively referring to diagnosable disorders. PTB adolescents exhibit a higher prevalence of developing psychiatric disorders suggesting increased emotional and psychological vulnerability. Frazier et al. [11] reported that 15% of PTB adolescents had received a diagnosis of one psychiatric disorder, 9% had been diagnosed with two co-occurring disorders, and 8% with three or more diagnoses, resulting in a total of 33% of PTB adolescents having been diagnosed with at least one psychiatric disorder. This prevalence exceeds global estimates for diagnoses of psychiatric disorders in the general population of children and adolescents considerably, which range between 10 and 20% [62]. Furthermore, significant sex differences were reported, with girls being more frequently diagnosed with major depressive disorder (6% vs. 2%; *OR* = 3.33, *p* <.05), generalised anxiety disorder (11% vs. 5%; *OR* = 2.5, *p* <.01), agoraphobia (7% vs. 1%; *OR* = 5, *p* <.001), separation anxiety disorder (6% vs. 2%; *OR* = 3.33, *p* <.01), and social anxiety disorder (8% vs. 3%; *OR* = 3.33, *p* <.01), but less often with the hyperactive subtype of ADHD (1% vs. 4%; *OR* = 0.24, *p* <.05; [11]).

Attention deficits and hyperactivity deserve specific attention within PTB adolescents, with ADHD being particularly prevalent (7–23%) and clinically relevant [11]. Research findings indicate that parent and teacher reports support the presence of more frequent ADHD symptoms in PTB compared to FT peers. Furthermore, while attentional problems were reported by 25% of the PTB adolescents aged 14, ADHD was diagnosed in only 7% of the included PTB adolescents [52]. In addition, at age 15, 18% of the PTB adolescents met criteria for ADHD, with a significant sex difference in the combined subtype (*p* =.004), characterised by both inattentive and hyperactive-impulsive symptoms and the hyperactive subtype being more common in boys than girls (*p* <.001; [11]). Among those diagnosed with autism spectrum disorders at age 10, 46.2% also met criteria for ADHD [11]. Interestingly, 61.5% of girls with autism spectrum disorder had comorbid anxiety disorders—more than double the rate compared to girls without ASD (26.7%; [11]).

Comparing PTB and family vulnerability hypotheses of attention deficits, we find a higher prevalence of ADHD in unrelated individuals (*β* = 0.64, 95% CI [0.17, 2.05], *p* =.04; [55]) compared to siblings (*β* = 0.32, 95% CI [0.05, 0.58], *p* =.01; [55]). This result suggests that shared genetic or familial factors may not account for the observed comorbidity. Similarly, PTB individuals were more likely to receive an ADHD diagnosis than their siblings (*p* =.01), providing evidence for a specific vulnerability due to PTB circumstances [35].

While attention deficits and hyperactivity are prevalent in PTB adolescents, engaging in physical activity might play a protective role in their mental health. Specifically, increased physical activity was linked to greater psychological well-being (*β* = 0.05, 95% CI [0.019, 0.087]; [34]) and more favourable self-perception (*β* = 0.06, 95% CI [0.028, 0.094]; [34]).

The findings emphasise the considerably increased risk of PTB adolescents to develop neurodevelopmental or psychiatric disorders, evidenced by elevated rates of psychiatric diagnoses and comorbidities. Pronounced gender disparities are also noteworthy. Of note is the high prevalence of attention deficits and hyperactivity in PTB individuals, which frequently co-occur with other neurodevelopmental conditions. This overlap complicates isolating the specific developmental pathways and contributions to mental ill-health. Thereby, it constitutes a potential confounding factor in studies aiming to assess the long-term impact of prematurity on cognitive and psychiatric outcomes that warrants attention.

## Discussion

The present systematic review aimed to investigate (1) whether evidence for a possible interplay between preterm birth and cognitive, visual, and motor abilities exists, and (2) whether and how these factors may relate to mental health outcomes of PTB individuals in adolescence. The reviewed findings provide evidence for PTB individuals exhibiting independent differences in some of the reviewed domains. Specifically, we found lower performance on cognitive tests in early adolescence, which re-appear in late adolescence as individuals approach adulthood. As well, the results reveal a higher prevalence of psychiatric disorders, such as ADHD, anxiety, and autism, in PTB populations [9, 11, 33, 34, 55]. Evidence for general motor difficulties, excluding NSI and cerebral palsy, and subtle visual aberrancies, excluding ROP and CVI, is less ubiquitous. Direct links between all reviewed domains and mental health are scarce. The only identified study links cognition to mental health [11].

The presented meta-analyses demonstrate comparable reductions in IQ in the beginning and end of adolescence, with little difference in cognitive performance during the height of neural rewiring in mid-adolescence [35]. This suggests that cognitive abilities are significantly reduced at two time points with major life changes (i.e., start of adolescence and reaching legal adult age). Importantly, increasing differences from age 15 to 19 may be suggestive of persisting differences in cognitive functioning going into adulthood [44, 47]. More specific cognitive aspects showing deficits in PTB adolescents include executive functions, attention, and planning. These specific difficulties became evident in standardised testing and parental reports, and can significantly impact everyday executive functioning [50, 53]. Similar cognitive deficits in 11-year-old PTB individuals, observed across two separate samples born a decade apart, indicate that these impairments persist despite advancements in neonatal care in the meantime [45].

From a mental health perspective, we found a substantially increased prevalence of psychiatric disorders in PTB populations [3, 11, 24, 33–35, 47, 48, 54, 63]. Across publications ADHD, anxiety, and autism were most elevated [9, 11, 33, 34, 55]. These findings on prevalence receive support from recent WHO reports showing an increased risk of developing similar psychiatric disorders including ADHD, anxiety, and affective disorders by PTB individuals [6, 9, 55]. A potential driver of more frequent internalising problems may be found in increasing screen use (i.e., social media and smartphones) of adolescents [64, 65]. It may even be conceivable that gender plays a role for the level of internalising problems, as one of the reviewed studies [11] reports a higher risk for female PTBs to develop internalising problems. Gender differences are supported by general trends of increased needs for mental health care by US girls [66] and elevated internalising problems in female adolescents [64]. It is also conceivable that environmental [67], genetic [68], and familial factors [69] contribute to the increased vulnerability of PTB individuals to develop psychiatric disorders. Though, a detailed analysis and discussion of these aspects was out of scope for the present review.

We also identified evidence for reduced motor skills in PTB adolescents. For instance, fine motor impairments are pronounced in PTBs, with adolescents showing lower performance on tasks such as the RCFT [50]. The majority of PTB individuals aged 10 also showed performance considerably below average in visuo-motor precision—another fine motor task [42]. Although cerebral palsy, a condition we excluded, was part of some reviewed studies’ definition of NSI [33, 34], reduced motor skills also hold for the substantial proportion of PTB adolescents without NSI [33]. These differences in addition to more severe functional limitations, including NSI [70] and cerebral palsy [71], underline the motor challenges faced by PTB adolescents. Reduced motor skills in PTB individuals may be explained by partially altered cerebellar development [72, 73]. These alterations likely result from the disrupted neuronal proliferation and differentiation, particularly in this brain region, during the last trimester of pregnancy [74], and can persist into adolescence and adulthood [50, 58].

Large-scale cohort studies [34], however, indicate that motor deficits are not universal among PTB individuals, highlighting the heterogeneity of outcomes in this group. This variability in motor outcomes may stem from the heterogeneous nature of PTB populations and the individual birth insults and negative factors they experience. Although many studies report the number of PTB participants with NSI or cerebral palsy, they only present group-level results, which makes differentiation impossible. This limitation hinders our ability to draw more generalisable conclusions about the nature of motor deficits across the diverse PTB population.

Similar to the literature on motor abilities, PTB samples are often heterogeneous. Therefore, we took a closer look at study quality and heterogeneity across outcomes. The quality assessment based on the STROBE criteria confirmed good to excellent methodological rigor across the included studies. Differences in gestational age between samples may systematically drive heterogeneity, as the particular influence of two studies in leave-one-out analyses of IQ scores suggested [35, 47]. The stronger deficits reported by Linsell and colleagues [47] likely reflect their focus on extremely preterm adolescents (< 26 weeks), whereas James et al. [35] found smaller deficits in a study including late preterm participants (up to 37 weeks). The observed heterogeneity within both IQ meta-analyses also indicates a multifactorial nature of heterogeneity across studies, given that the number of studies included in these meta-analyses was small, the age range of participants at birth and testing wide (capturing almost all preterm and adolescent age groups), and the variation in study settings, countries, and assessment tools considerable.

We also considered potential cohort effects related to advances in medical care or education. When ordering studies by birth and sampling year, we find no significant correlative relations with IQ differences over time. This result suggests that cohort effects are unlikely to account for the observed variability alone. Thus, no single factor can be pinpointed as the driver of the observed heterogeneity in IQ scores between studies. The core finding of reduced cognitive performance in PTB adolescents remains consistent across analyses and robust to sensitivity testing.

Similar to cognitive outcomes, heterogeneity in mental health and visual or visuo-motor outcomes is likely driven by the use of a variety of assessment tools, including self- and parent-reports, routine clinical data, and measures based on clinical diagnosis or screening. Most of these data are typically derived from help-seeking populations. Despite this variety in methods, the available evidence converges on indicating a substantial prevalence of mental health diagnoses in a considerable number of adolescents after PTB. A meaningful investigation of heterogeneity in subtle visual outcomes will require more future research.

Here, we intended to focus on investigating functional visual abilities with rather less severe consequences that have not received much attention, due to the known early visual alterations manifesting in CVI in preterm populations, and may not differ at first sight. The identified studies investigating visual skills in PTB adolescents without CVI show subtle deficits in visual abilities. These include visual perception, visuo-motor control, and visuo-spatial planning, and can become more pronounced during adolescence [42, 75]. An effect on academic [76] and psychosocial functioning [77] in children and adolescents with visual impairments is possible as well. While these findings provide preliminary suggestive evidence for aberrant functional visual abilities in PTB individuals born without CVI, the small number of three reviewed studies indicates a clear gap in the literature. This gap underscores the need for future research on subtle visual-perceptual difficulties beyond ROP and CVI.

The limited evidence on visual abilities reviewed herein might have its reasons in excluding publications with participants suffering from CVI. We excluded these studies, since CVI encompasses a broad range of neuro-visual disorders [78], which complicates a clear differentiation of visual abilities in such studies. In this respect, many studies do not differentiate between more subtle and severe cases. For example, some have included a substantial number of PTB individuals with comorbid CVI or cerebral palsy [79] or more severe intraventricular haemorrhage (≥ grade III) in the same group analyses. Further, it is important to note that subtle visual-perceptual difficulties may go undiagnosed, inadequately assessed or undercorrected into adolescence [80], as the prevalence of suffering from severe visual deficits including ROP or CVI is substantial and may have drawn most of the attention and resources in the PTB context.

More generally speaking, accurate visual functioning is assigned importance, since its absence may have enduring effects on academic performance, daily life, and overall mental health [81, 82]. For instance, elevated rates of anxiety and depression have been reported among people with visual impairments [83] and links between developmental self-regulation and visuo-spatial skills [83, 84]. These general aspects suggest the importance of ongoing monitoring of the full spectrum of visual functioning in childhood and adolescence [78, 82, 83].

Whilst the present review provides separate evidence for cognitive and motor impairments and an increased risk of psychiatric disorders in PTB adolescents, the direct investigation of the interplay between these functional domains and mental health outcomes remains scarce in the literature [85]. Nevertheless, several studies have yielded indirect evidence in support of such associations. For instance, the ELGAN cohort study revealed that participants with multiple psychiatric disorders were more likely to encounter cognitive impairments and academic challenges [11]. In addition, Brylka et al. [34] found that physical activity was associated with enhanced self-perception and a reduction in peer-problems among PTB and FT adolescents. Similarly, Morris et al. [86] showed that PTB individuals aged 9 with anxiety disorders often present with comorbid motor, social, and cognitive difficulties. These observations align with global estimates indicating that preterm birth is linked not only to separate cognitive and motor impairments but also to a wide spectrum of neurodevelopmental and psychiatric challenges [87]. This suggests that psychiatric symptoms may be part of broader neurodevelopmental vulnerabilities in PTB individuals.

Together these links and the general cognitive challenges we identified in this review hint at a relation to mental ill-health. Supporting evidence comes from lower cognitive performance being associated with elevated psychopathology in the general population [62], with greater cognitive deficits in severe, long-lasting disorders such as schizophrenia and bipolar disorder [2, 88, 89]. In adults, this relationship appears to transcend boundaries of individual psychiatric diagnoses [90], which suggests that cognitive deficits are not confined to specific disorders but rather reflect a broader transdiagnostic phenomenon. This transdiagnostic view is endorsed by child and adolescent clinicians engaging with adolescent patients [91], which points to its potential for the heterogeneous group of PTB adolescents. It is important to note, however, that psychiatric outcomes in PTB adolescents are influenced by multiple factors beyond prematurity itself. Environmental influences [92], familial context [25], and genetic predisposition all contribute to the emergence of psychiatric symptoms [93], and PTB should be considered as one of several interacting risk factors rather than a sole causal agent.

Interestingly, deficits in motor skills can also negatively impact cognitive functioning and psychosocial factors in non-PTB populations. In school-age children up to 13 years, gross motor deficits have been associated with greater cognitive challenges and may impact academic performance [94–96]. Similarly, fine motor skills have been linked to cognitive performance [81], as they predicted higher academic achievement [97]. Moreover, language and motor function disorders have been associated with long-term effects on mental health and overall functioning that can persist well into adulthood [98, 99]. Finally, motor difficulties have been linked to psychosocial adjustment, emotional well-being, and reduced social engagement [87, 100, 101].

While the present systematic review offers valuable insights, some limitations warrant consideration. Firstly, limiting inclusion to cohorts born on the 1 st of January 1980 or later may have introduced selection bias. This timeframe, however, helps to capture extensive evidence on how preterm birth relates to the reviewed domains while reflecting advances in neonatal care. Thereby, it remains highly relevant to contemporary clinical and developmental contexts and strengthens the applicability of the conclusions to current PTB populations. Secondly, while heterogeneity in assessment methods and diagnostic criteria across the included studies complicates direct comparisons and more extensive meta-analyses, the review’s systematic methodology, incorporating small meta-analyses of studies of mostly excellent quality, enabled the most meaningful integration and synthesis of findings. Thirdly, our meta-analysis of IQ effect sizes shows one study reporting two substantially more reduced effect sizes. This could have biased the outcome of this meta-analysis. A complementary leave-one-out meta-analysis, however, showed consistent substantial reductions in IQ even without this study. As well, this study does not stand out in our complementary analysis of mean IQ differences either. These findings provide evidence against an exaggeration of the observed results. Fourthly, in the interpretation of findings on mental health outcomes one needs to keep in mind that data have been acquired through varying methods including self- and parent-reports, and routine clinical data. Most of these data may have been sourced from help-seeking populations and by using varying instruments for diagnostics. Fifthly, we excluded studies involving individuals with CVI or ROP. This criterion may have inadvertently resulted in an underrepresentation of visual-perceptual difficulties in the literature. Several publications report worse visual outcomes in relation to CVI and ROP, which can present in many ways. However, these studies were excluded as they do not differentiate samples into adolescents with more and less severe comorbid conditions. Hence, we aimed to capture samples with less severe impairments that have previously received little attention. Finally, most studies reviewed herein present evidence of differences in one domain, with direct links remaining limited. While this scarcity does not allow for causal conclusions on direct links in PTB populations, it should be considered as suggestive evidence underlining the need for more validating research. Additionally, it should be noted that most of the included studies were observational in nature, which also limits the ability to draw causal inferences and underlines the need for future longitudinal and experimental research to validate these associations.

Overall, the evidence for separate differences in the reviewed domains in PTB groups, in combination with bivariate links between these domains in individuals from the general population, addresses the aims of the present review. The findings have implications for research and clinical practice. Research on PTB adolescents should focus on the interactions between mental health, motor skills, and physical activity to clarify underlying mechanisms [34, 97, 102]. Investigations of visual and visua-motor functioning, even in the absence of CVI, should be incorporated, given the central role of vision in neural development and its influence on cognitive and psychiatric outcomes [15, 16, 103]. This addition could elucidate the full spectrum of potential challenges in visual functioning. For more mechanistic insights on the degree of motor difficulties and contributing factors, such as gestational age, birth complications, and variations in postnatal care, we recommend considering these aspects in future studies and dissociate results accordingly [103]. Longitudinal, multimodal studies combining cognitive, motor, visual, and neuroimaging data could be particularly valuable for mapping developmental trajectories and uncovering the mechanisms underlying long-term challenges in PTB individuals [58, 104]. Integrating genetic and neurobiological markers, such as prenatal gene expression and genetic risk scores, in future studies could further improve prediction of psychiatric outcomes [43, 105].

Clinical practice could benefit from the presented results by implementing routine screening of cognitive, motor, visual, and psychiatric domains in follow-up care and early detection programs. In this context, it may be valuable to include comprehensive evaluations of motor coordination and visual-perceptual abilities, allowing for the early identification of subtle neurodevelopmental difficulties that could precede or co-occur with psychosocial challenges in preterm-born adolescents. This recommendation is supported by PTB adolescents being at an increased risk for psychiatric conditions and these frequently show early signs during adolescence [2, 4, 34, 51]. Cognitive and visuo-motor evaluations could be included in screenings to support academic and functional outcomes due to enduring difficulties in cognition [53, 106]. To improve long-term outcomes, drawing on and potentially merging established prevention frameworks, such as selective and indicated prevention [55, 106], may offer valuable opportunities to strengthen early detection efforts. Although our results inevitably raise the question of how much redundancy exists between the reviewed abilities that could be exploited in future screening approaches, tailoring early interventions to individual multidomain profiles might address the complexity of PTB profiles and serve those with overlapping difficulties in particular.

In conclusion, this systematic review highlights that PTB adolescents face mental health challenges more often. We also show that cognitive and motor development are likely impacted by being born PTB, with differences persisting into adolescence and even adulthood. Notably, research on long-term subtle functional visual abilities in PTB adolescents, beyond ROP and CVI, and on direct links between the reviewed domains is still limited. More studies are needed that systematically address visual functioning and motor outcomes, and their interplay with mental health, to better understand developmental mechanisms and inform future screening and intervention approaches.

## Data Availability

All data generated or analysed during this study are included in this published article. Additional materials related to the meta-analyses (e.g., extracted data tables, risk of bias assessments, and supplementary analyses) are available via the Open Science Framework repository: https:/osf.io/me2d6.
